# Immune activated monocyte exosomes alter microRNAs in brain endothelial cells and initiate an inflammatory response through the TLR4/MyD88 pathway

**DOI:** 10.1038/s41598-017-10449-0

**Published:** 2017-08-30

**Authors:** Pranjali Dalvi, Bing Sun, Norina Tang, Lynn Pulliam

**Affiliations:** 1 Department of Laboratory Medicine, Veterans Administration Medical Center, San Francisco, CA USA; 2 Departments of Laboratory Medicine and Medicine, University of California, San Francisco, CA USA

## Abstract

The host immune response is critical for homeostasis; however, when chronic low level activation of the immune response with or without the driver continues, a cascade of events can trigger immunological dysfunction. Monocytes are key peripheral sensors of the immune response and their activation is instrumental in the development of cognitive impairment. Here, we show that monocytes activated by interferon alpha, lipopolysaccharide or a combination of both generate exosomes carrying significantly altered microRNA profiles compared to non-activated monocytes. These exosomes alone can activate human brain microvascular endothelial cells to stimulate adhesion molecules, CCL2, ICAM1, VCAM1 and cytokines, IL1β and IL6. This activation is through the toll like receptor 4 (TLR4)/myeloid differentiation primary response gene 88 (MyD88) pathway that activates nuclear factor-κB and increases monocyte chemotaxis. Inhibition of monocyte exosome release reverses endothelial cell activation and monocyte chemotaxis. Our study suggests that activated monocytes have an impact on brain vascular function through intercellular exosome signaling.

## Introduction

Exosomes have emerged as a new class of bio-nanoparticles that have been recognized to potentially change the face of physiological and pathological conditions in humans. Exosomes are defined as 40 to 100 nm sized extracellular vesicles that result from the fusion of multivesicular bodies with the plasma membrane^1^. We recently predicted an inflammatory disease mechanism in which human umbilical vein endothelial cells (HUVECs) actively expressed chemokine (C-C motif) ligand 2 (*CCL2*), interleukin 6 (*IL6*) and intercellular adhesion molecule 1 (*ICAM1*) genes after exposure to exosomes derived from interferon alpha (IFNα), lipopolysaccharide (LPS) or IFNα followed by LPS (I/L) stimulated monocytes^2^. This occurs via the activation of the toll like receptor 4 (TLR4)/Myeloid differentiation primary response gene 88 (MyD88)/nuclear factor-κB (NF-κB) pathway.

Lipopolysaccharide, the endotoxin of microbial origin, is well known to trigger inflammation in the infected host. Inflammation causes disruption of the blood brain barrier (BBB) leading to numerous diseases including multiple sclerosis, Alzheimer’s disease and stroke^3^.

Similarly, prolonged IFNα activity during chronic viral infections can have deleterious effects on cognitive function^4, 5^. IFNα endogenously produced during hepatitis C virus (HCV) or hepatitis B virus infections or exogenously introduced for the treatment of such common viral infections causes neuropsychiatric disorders^6^. IFNα treatment for HCV has also been reported to cause systemic lupus erythematosus^7^. Additionally, many have reported that IFNα therapy used for treating cancers can initiate rheumatoid arthritis^8, 9^. Inflammation consequently activates TLR4 and NF-κB in bystander cells, thereby leading to the production of cytokines, adherence factors and chemokines in these cells^2^.

We and others have reported that microRNA (miR)-146a/b and miR-155 promote endotoxin-mediated inflammation in endothelial cells^2, 10^. Most of these regulatory microRNAs (miRNA) are produced in innate immune cells upon their activation^11^. The miRNAs are then internalized by the multivesicular bodies in the immune cells and subsequently get packaged into exosomes, which carry these miRNAs to the surrounding cells. Thus, the recipient cells show increased expression of these miRNAs consequently triggering immune dysfunction related proinflammatory pathways^10^.

Human brain microvascular endothelial cells (HBMECs) are major components of the blood brain barrier. The HBMEC lining is responsible for limiting the passage of soluble substances and cellular components from blood to the brain^12^. An increase in inflammatory cytokines, chemokines and adherence factors in HBMECs would disrupt this barrier, causing leakage of undesirable molecules into the brain. Neuropathogenesis and dementia due to the transmigration of activated human immunodeficiency virus (HIV)-infected monocytes and macrophages across the BBB is an example of how excessive production of chemoattractants in HBMECs could facilitate this migration^13^.

We report here that exosomes derived from activated immune cells are responsible for carrying proinflammatory contents including miRNAs to the brain via the brain endothelium thereby promoting monocyte chemotaxis. Moreover, we show that the prevention of exosome release from these activated monocytes could completely prevent the increase of inflammatory molecules on brain endothelial cells. Our results support the need for further investigating exosome technology as a treatment option for immune initiated pathologies.

## Results

### Activated monocyte exosomes enhance chemotaxis

**Figure 1:**
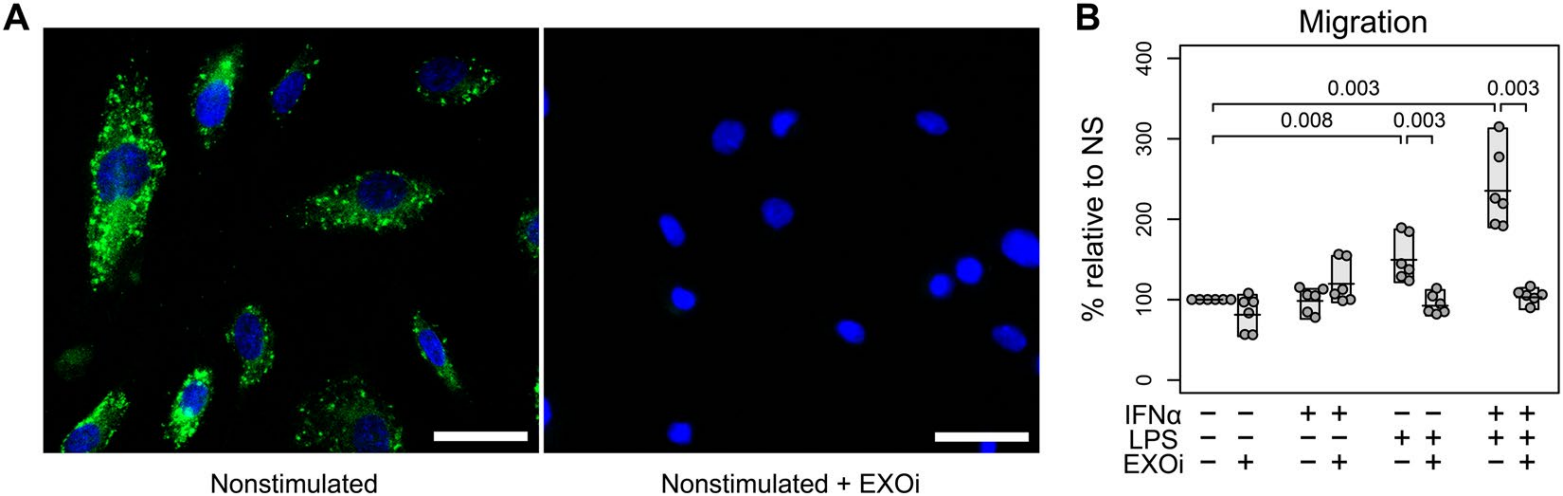
Exosomes from LPS and I/L activated monocytes increase migration towards brain endothelial cells. (**A**) HBMECs receiving exosomes from calcein AM dye (green) stained, nonstimulated human monocytes in the upper-well (left panel). Monocytes were treated or not with the exosome inhibitor GW4869 (EXOi) (right panel). Scale bar: 50 µm. Representative picture of triplicates. (**B**) Migration of activated monocytes toward HBMECs was quantified and compared to that of monocytes treated with EXOi (n = 6). Shaded boxes indicate range of the data, horizontal bars indicate mean. Two-sided paired Student’s *t* tests with multiple comparison correction were used.

We first wanted to determine that HBMECs take up exosomes from calcein AM stained monocytes on top of a dual chamber system. We observed punctate green fluorescent exosomes throughout the HBMECs within the first 3 hours of coculture (Fig. 1A, left panel). This indicated that the exosomes released from the fluorescently labelled monocytes were stained as well and were taken up by the HBMECs in the bottom chamber. As expected, the HBMECs cocultured with monocytes that were incubated with exosome inhibitor, GW4869 (EXOi) showed no green fluorescence (Fig. 1A, right panel). This confirmed that GW4869 inhibited sphingomyelinase 2 in the monocytes that stopped the budding of exosomes from multivesicular bodies.

Exosome trafficking to the HBMECs was significantly enhanced when the monocytes in the upper chamber were activated with LPS or I/L (Fig. 1B). This was evident from the lack of migration of LPS or I/L stimulated monocytes in the presence of the exosome inhibitor, GW4869. The maximum increase in monocyte migration was seen with the combined I/L stimulation. The monocyte stimulation with IFNα alone did not cause an increase in their migration (Fig. 1B). Also, there was no difference in the migration rates of nonstimulated monocytes with or without the inhibitor. Overall, these data show that the LPS or I/L stimulation was responsible for generating exosomes carrying a different molecular cargo, which facilitated their migration towards endothelial cells.

### Monocyte exosomes stimulate cytokines and adhesion molecules in brain endothelial cells

**Figure 2:**
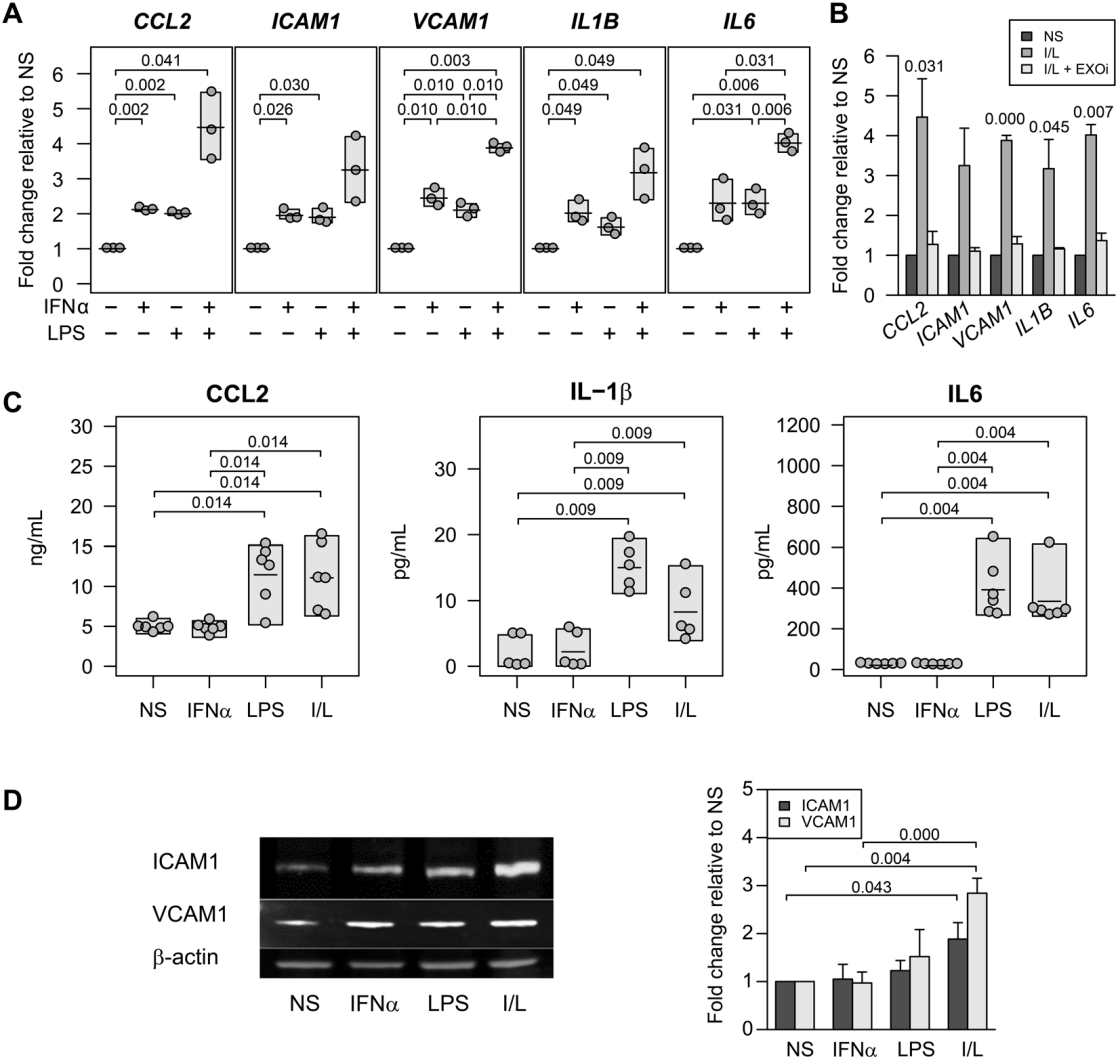
Increase in brain endothelial cell activation is due to monocyte derived exosomes. (**A**) HBMECs were cocultured with exosomes derived from IFNα, LPS or I/L activated monocytes. Selected genes were analyzed by real time qPCR (n = 3). (**B**) I/L activated monocytes were incubated with or without exosome inhibitor (EXOi) and cocultured with HBMECs in a cell culture insert. qPCR was performed on HBMECs after 3 h (n = 3), nonstimulated﻿ (NS) and I/L are the same samples as in Fig. 2A. (**C**) ELISA from conditioned media of HBMECs (n = 5 or 6) cocultured with exosomes derived from NS, IFNα, LPS or I/L activated monocytes. (**D**) Western blot of HBMECs exposed to exosomes from NS, IFNα, LPS or I/L activated monocytes. The blots are a representative of four experiments. The bar graph shows the average densitometry analysis using ImageJ software (n = 4). The shaded boxes in (**A**) and (**C**) represent the range and the horizontal bars in each box is the mean. Quantitation data in (**D**) are presented as mean ± s.d. Two-sided paired Student’s *t*-tests with multiple comparison correction were used.

We previously published that human umbilical vein endothelial cells treated with LPS and I/L stimulated monocyte derived exosomes showed increased mRNA and protein expression of ICAM1, CCL2 and IL6^2^. We tested whether the HBMECs are susceptible in a similar manner. The mRNA expression of chemokines *CCL2*, *ICAM1*, vascular cell adhesion molecule 1 (*VCAM1*) and cytokines interleukin 1 beta (*IL1B*) and *IL6* in HBMECs cocultured with I/L stimulated monocytes was significantly increased compared to nonstimulated, IFNα or LPS stimulated monocytes (Fig. 2A). The *CCL2*, *ICAM1* and *VCAM1* mRNA were significantly increased in HBMECs cocultured with IFNα stimulated monocytes compared to nonstimulated. *CCL2*, *ICAM1*, *VCAM1* and *IL6* mRNA were significantly upregulated in HBMECs cocultured with LPS stimulated monocytes to a similar degree as IFNα. The key role of exosomes in activating the transcription of these molecules was established from the fact that the HBMECs cocultured with monocytes treated with the exosome inhibitor GW4869, along with I/L stimulation did not show any increase in mRNA of the adherence factors or inflammatory markers (Fig. 2B). In addition, we also observed a significant increase at the translational level of CCL2, IL6 and IL1β proteins using the conditioned media from HBMECs treated with LPS or I/L stimulated monocyte exosomes compared to nonstimulated or only IFNα stimulated monocyte exosomes (Fig. 2C). Similarly, there was a significant increase in the protein expression of adherence molecules ICAM1 and VCAM1 in HBMECs exposed to I/L monocyte exosomes seen by western blot and quantified by densitometry analysis (Fig. 2D). These data indicate a significant role of monocyte exosomes in activating chemokines and cytokines in the bystander brain endothelial cells.

### I/L stimulated monocytes and exosomes have a distinct microRNA expression profile compared to IFNα or LPS alone

**Figure 3:**
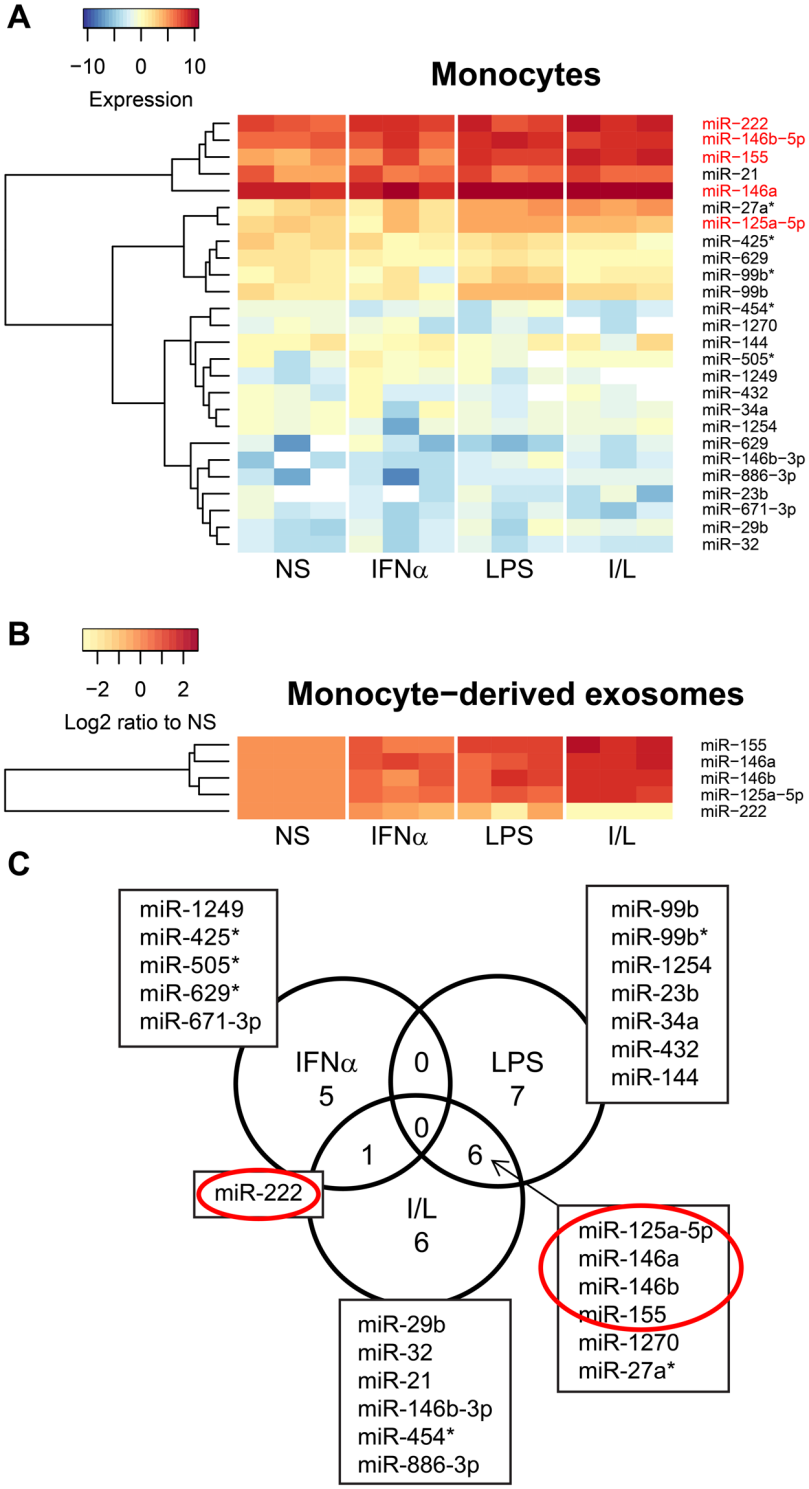
miRNAs are significantly modulated by IFNα and LPS stimulation of monocytes. (**A**) miRNA arrays were performed on normal human monocytes isolated from blood and stimulated with IFNα, LPS or both (I/L) (n = 3). Differentially expressed genes between groups are shown. Full heatmap is shown in supplemental Figure S1. (**B**) Heatmap of selected miRNA expressions from monocyte derived exosomes (n = 3) using real time qPCR. (**C**) Venn diagram representing differentially up or down regulated monocyte miRNAs overlapping between the groups as shown in (**A**). Red circles show selected miRNAs of interest.

Since exosomal miRNAs have a crucial role in influencing the inflammatory response in recipient cells^10^, we analyzed the miRNA profile of monocytes from all 4 treatments (Fig. 3A). A complete list of all monocyte miRNAs analyzed is presented in Supplement Fig. S1. Specifically, the expression of miR-222, miR-155, miR-146a, miR-146b and miR-125a-5p increased with single IFNα or LPS treatment (Fig. 3A). However, with dual I/L treatment, this increase was maximum. For further investigation based on our previous results^2^ and literature pertaining to miRNAs regulating inflammation upon immune stimulation^10, 14–16^, we analyzed monocyte exosome miRNAs by quantitative real-time polymerase chain reaction (qPCR) (Fig. 3B). This profile differed between monocytes and exosomes. miR-222 expression showed the opposite results in I/L monocytes (1.3 fold increase compared with NS) and their derived exosomes (5.5 fold decreased compared to NS). While there was a constitutive expression in nonstimulated monocytes, miR-222 significantly increased with I/L stimulation. Interestingly, this miRNA did not get transferred to the I/L monocyte exosomes (Fig. 3B). Another striking observation was the enhanced packaging in I/L monocyte exosomes compared to the monocyte parent cell of miR-125a-5p (2.5 fold vs 1.3 fold), miR-146a (3.2 fold vs 1.2 fold), miR-146b (2.8 fold vs 1.3 fold) and miR-155 (3.7 fold vs 2.0 fold) (Fig. 3B). These data point to the unique role of exosomes in causing specific physiological changes in the microenvironment in which they are released in stress conditions which, in this case is I/L. We checked for miRNAs commonly up or down-regulated in monocytes between the three treatment groups. As shown in the Venn diagram (Fig. 3C), miR-222 expression increased in both IFNα and I/L groups. miR-125a-5p, miR-155, miR-146a, miR-146b and miR-27a* increased in the LPS and I/L groups, while miR-1270 was decreased. There were no common miRNAs differentially regulated between the IFNα or LPS group in this dataset (Fig. 3C). This indicates that these individual immune modulators function very differently in immune cells compared to their combination.

### Monocyte exosomes transfer functional miRs to cocultured HBMECs

**Figure 4:**
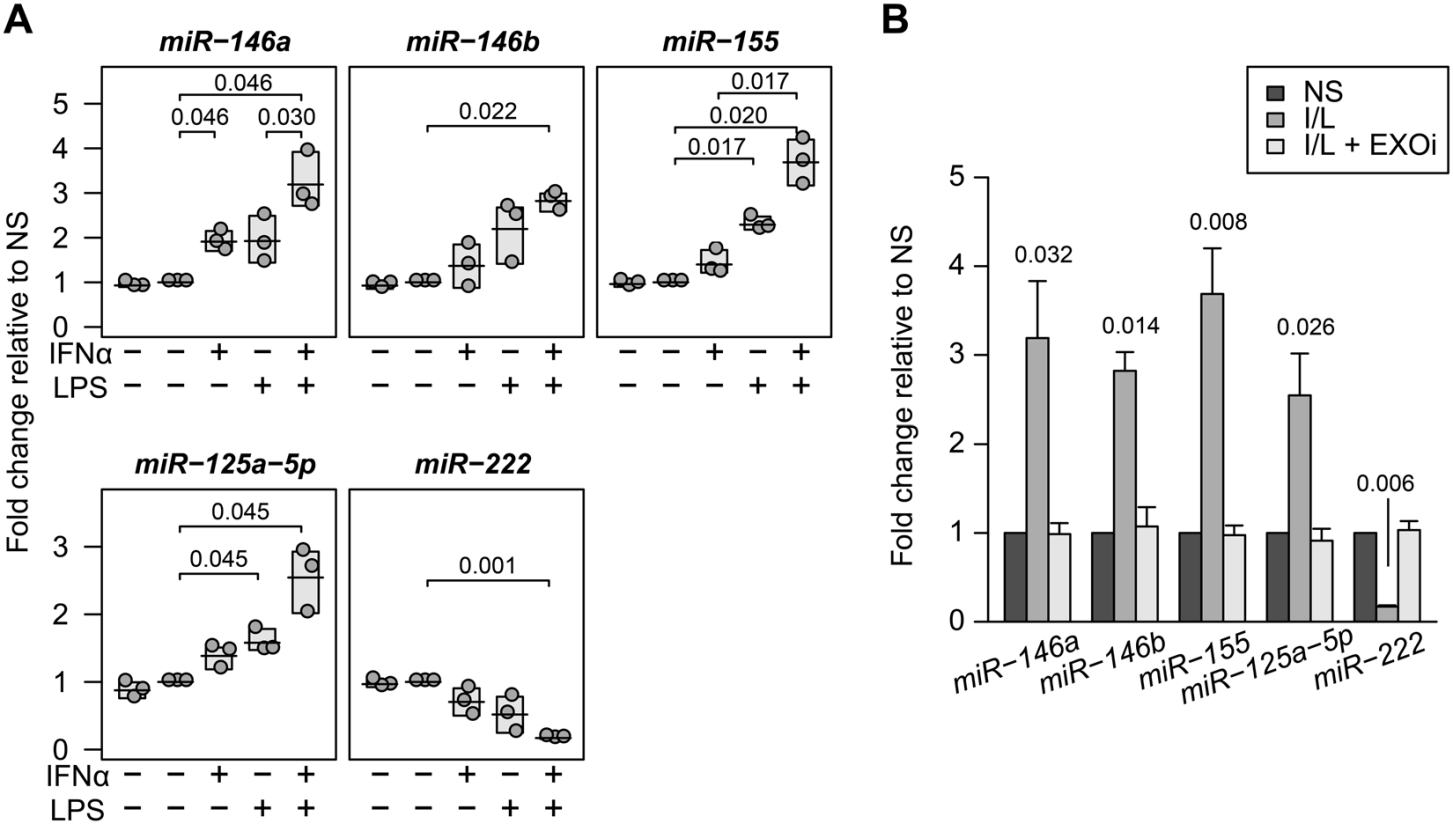
Monocyte derived exosomes transfer functional miRNAs to HBMECs. (**A**) HBMECs were co-cultured with exosomes derived from IFNα, LPS or I/L activated monocytes. HBMECs were analyzed by real time qPCR for selected miRNAs. The first treatment group in every graph represents HBMECs without exosomes. The second treatment group represents HBMECs treated with nonstimulated exosomes. Each dot represents the mean of technical triplicates. The shaded boxes represent the range and the line in each box is the mean ﻿of ﻿the group. (**B**) I/L stimulated monocytes were incubated with HBMECs in the presence or not of exosome inhibitor (EXOi) in the upper-well of a dual chamber cell culture system. HBMECs were analyzed by real time qPCR for selected miRNAs. NS, nonstimulated. Experiments were performed in triplicate for each of 3 different blood donors. Data in (**B**) are presented as mean ± s.d. Two-sided paired Student’s *t*-tests with multiple comparison correction were used.

Since the miRNAs differentially expressed in I/L stimulated monocyte exosomes are known to be involved in regulating inflammation or adhesion markers in endothelial cells^17–19^, we sought to determine whether they are transferred to HBMECs. HBMECs treated with nonstimulated exosomes (second treatment group) had similar miRNA levels to HBMECs alone (first treatment group) in all miRs tested (Fig. 4A). However, miR-146a, miR-146b, miR-155 and miR-125a-5p were significantly increased in HBMECs cocultured with I/L stimulated monocyte exosomes (Fig. 4A). The expression of miR-146a also significantly increased in HBMECs cocultured with IFNα stimulated monocytes, whereas miR-155 and miR-125a-5p increased significantly in HBMECs incubated with LPS stimulated monocytes. The expression of miR-222 was significantly down regulated in HBMECs cultured with I/L stimulated monocytes. All these changes in miRNA expression were attributed to monocyte exosomes, since the presence of inhibitor GW4869 in the upper chamber containing the monocytes completely normalized the miRNA expression in HBMECs to the level of cells cultured with nonstimulated monocytes (Fig. 4B). These data confirmed the direct contribution of exosomes in transferring functional miRNAs from parent monocytes to the recipient HBMECs.

### Monocyte exosomes activate the TLR4/MyD88/NF-κB signaling pathway in HBMECs leading to differential miRNA expression

**Figure 5:**
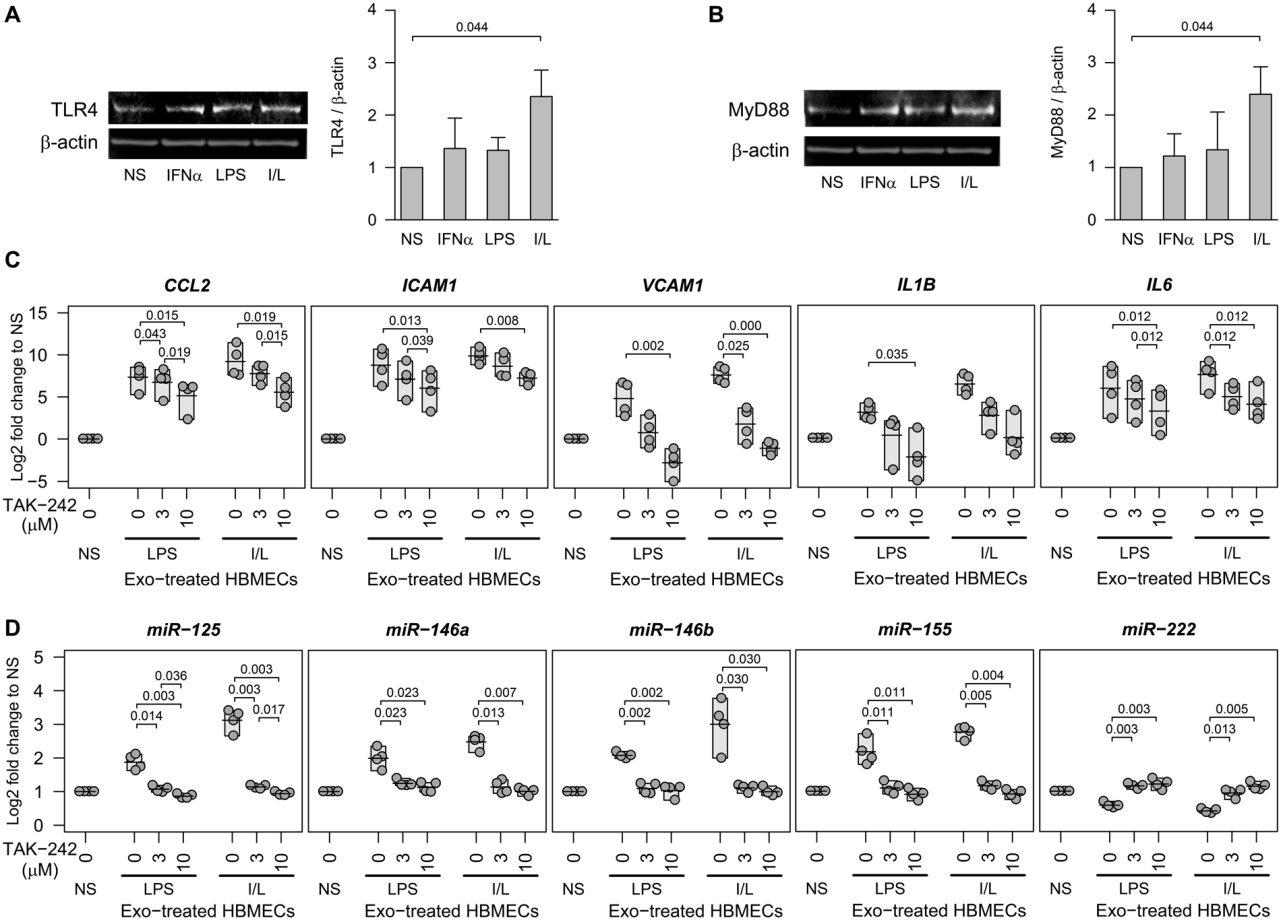
Inhibition of TLR4 reduces exosome mediated adhesion molecules/cytokines and miRNAs in a dose dependent manner and normalizes differential miRNA transcription. IFNα and/or LPS treated monocyte exosomes were added to HBMECs followed by representative western blots for (**A**) TLR4 and (**B**) MyD88. Graphs represent densitometry analysis (n = 3). (**C** and **D**) HBMECs were pretreated with various doses of TLR4 inhibitor, TAK-242. Nonstimulated (NS), LPS or IFNα and LPS (I/L) treated monocyte exosomes were added to HBMECs for 24 h. qPCR was performed in triplicate for each of 4 different blood donors. Each dot represents the mean of the triplicate. The shaded box represents the range and the horizontal line is the mean of the group. Two-sided paired Student’s *t* tests with multiple comparison correction were used. For repeated measures, Page’s trend tests showed all gene expressions had a decreasing trend with increasing dose of TAK-242 in both LPS and I/L exosome treated HBMECs (*P* < 0.01 for all genes, except *IL1B* and *IL6* with I/L-exosome treatment were *P* < 0.05).

We previously reported that inhibiting the NF-κB in HUVECs treated with exosomes from LPS or I/L stimulated monocytes significantly reduced the elevated expression of *ICAM1* and *CCL2* but not *IL6* ^2^. Additionally, there are numerous reports stating the presence of NF-κB binding sites on miR-146, miR-155 and miR-222. Therefore, we first checked whether the I/L stimulated monocyte exosomes stimulate the TLR4/MyD88 pathway, thereby activating NF-κB. HBMECs incubated with exosomes from I/L treated monocytes showed significant increases in the protein expression of TLR4 (Fig. 5A) and MyD88 (Fig. 5B).

**Figure 6:**
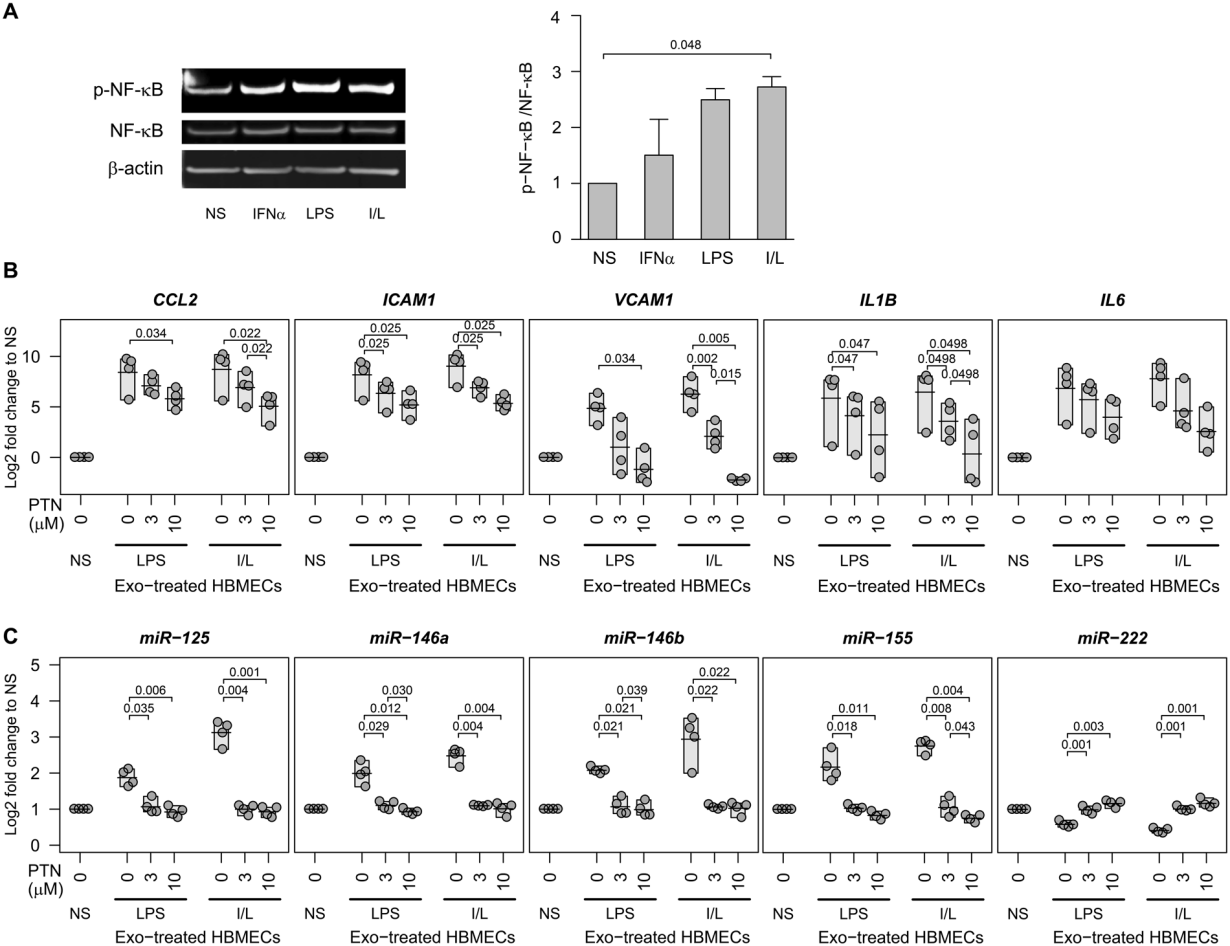
Inhibition of NF-κB reduces exosome mediated adhesion molecules/cytokines and miRNAs in a dose dependent manner. (**A**) HBMECs were incubated with or without exosomes derived from nonstimulated (NS) or stimulated (IFNα, LPS, I/L) monocytes, and analyzed by western blot (n = 2). The graph represents densitometry analysis for protein expression. (**B** and **C**) HBMECs were pretreated with NF-κB inhibitor PTN. Exosomes derived from nonstimulated (NS) or stimulated monocytes (LPS, I/L) were added for 24 h. qPCR was performed in triplicates for each of 4 different blood donors. Each dot represents the mean of the triplicate. The shaded box represents the range and the horizontal line is the mean of the group. Two-sided paired Student’s *t* tests with multiple comparison correction were used. For repeated measures, Page’s trend tests showed all gene expressions had a significant decrease with increasing PTN dose in both LPS and I/L exosome treated HBMECs (*P* < 0.01 for all genes, except *CCL2* and *VCAM1* with LPS-exosome treatment were *P* < 0.05).

Similarly, the dual I/L treated monocyte exosomes caused significant phosphorylation of NF-κB p65, with no change in the total NF-κB protein level in HBMECs compared to LPS, IFNα or non-stimulated monocyte exosomes (Fig. 6A). When we inhibited TLR4 using small molecule inhibitor TAK-242 (Fig. 5C) or inhibited NF-κB using parthenolide (PTN) (Fig. 6B) to confirm the involvement of these pathways in exosome mediated chemokine/cytokine activation in HBMECs, we found a significant dose-dependent reduction in the abnormally raised levels of *CCL2*, *ICAM1* and *VCAM1* genes in LPS or I/L stimulated monocyte exosome treated HBMECs (Fig. 5C and Fig. 6B). Similarly, we observed a significant decrease in the elevated expression of *IL6* in I/L monocyte exosome treated HBMECs on inhibiting TLR4 (Fig. 5C). However, there was no significant normalization in the up-regulated *IL6* level on inhibiting NF-κB (Fig. 6B). Another noteworthy observation was the opposite effect on *IL1B* compared to *IL6*. NF-κB inhibitor had significant effect on I/L monocyte exosome treated HBMECs in terms of stabilization of *IL1B* expression, whereas the TLR4 inhibitor had no significant effect (Fig. 5C and Fig. 6B). The reason for this difference of effect on *IL1B* and *IL6* expression is most likely that although TLR4 upregulates both downstream target genes *IL6* and *IL1B*, *IL6* is activated through p38 mitogen-activated protein kinase (MAPK)/extracellular-signal-regulated kinase (ERK)/c-Jun N-terminal kinase (JNK) arm of the TLR4/MyD88 signaling, whereas *IL1B* is transcribed after NF-κB-p65 nuclear translocation^20^. The exosomes from non-stimulated monocytes had no effect on TLR4/MyD88/NF-κB activation in HBMECs.

To further clarify the role of this pathway in miRNA regulation, we assessed the levels of miR-125a-5p, miR-146a/b, miR-155 and miR-222 on TLR4/NF-κB inhibition in LPS and I/L stimulated monocyte exosome treated or untreated HBMECs. We found that these up/down regulated miRNAs were normalized to the endogenous level seen in non-stimulated exosome treated HBMECs (Figs 5D and 6C).

These data highlight the essential contribution of LPS and I/L stimulated monocyte exosomes in initiating TLR4/NF-κB signaling in HBMECs leading to hyperactivation of inflammatory genes and abnormal regulation of miRNAs associated with inflammation.

## Discussion

Exosomes are the body’s own blueprint that change per its disease state. It is well established that exosomes act as messengers of “alarm” signals that can be used to predict serious diseases^21, 22^. An important question is whether exosomes are the exclusive carriers of these alarm signals from their stressed parent cells to healthy cells in their microenvironment. Here we show that cytokine and endotoxin stimulated monocytes, that have the potential biomolecules for triggering inflammation and adherence factors, deliver their proactive content into HBMECs exclusively via exosomes. After delivery, migration of monocytes towards HBMECs increases along with increased inflammatory cytokines and adherence molecules in these cells. Importantly, if exosomes are not secreted by the activated monocytes, the HBMECs are unaffected.

The HBMECs are the main components of the BBB that limit the entry of cells and compounds into the brain. The disruption of the BBB by injury to the brain endothelium initiates a number of disease processes by recruitment of immune cells, including Parkinson’s disease, Alzheimer’s disease, epilepsy and inflammation of the central nervous system to name a few^23, 24^. One of our unique observations is that stimulated monocytes do not adhere to HBMECs, but rather the transfer of their miRNA content to HBMECs through exosomes initiates adherence. We posit that LPS and IFNα together quickly mobilize the monocyte exosomes to initiate a breach in the BBB via miRNA- mediated activation of proinflammatory molecules and adherence factors. Treatment of HUVECs with IL6 has been shown to increase ICAM1, VCAM1 and E-selectin protein expression enhancing the binding of CD4^+^ lymphocytes but not monocytes to these cells^25^. Our results indicate a similar yet novel mechanism illustrating direct activation of IL6 in endothelial cells through monocyte exosomes.

The significance of exosome transferred miRNAs has recently gained recognition. Exosomes deliver viral miRNAs in a more stabilized manner since the embedded miRNAs, being surrounded by a lipid bilayer, are protected from degradation by RNases in the blood^26^. This phenomenon is a double-edged sword as it can be exploited to stably deliver miRNAs or other genes into targeted cells by labeling engineered exosomes with dual markers: cell specific and anti-disease specific. On the other hand, the fact that the exosomes are more stable carriers of their RNA/protein content makes them pathogenic molecules, competently delivering undesirable components to normal cells. Thus, they potentially promote the spread of almost all known diseases^27–30^.

Certain miRNAs have been reported to be immunomodulatory including miR-155 and miR-146a^10^. These miRNAs reside in immune cells naturally, as observed in our miRNA arrays as well as by others^18, 31, 32^. The transfer of miR-155 from LPS-activated donor to recipient dendritic cells can promote inflammation^10^. Furthermore, miR-155 was reported to negatively regulate BBB integrity^23, 33, 34^. In a recent study on cerebral malaria, miR-155^−/−^ mice were shown to have improved BBB integrity and survival compared to wild type mice in spite of high peripheral parasitemia, further supporting our findings^34^. In the same study, the authors showed distinctly reduced vascular leakage in HUVECs incubated with sera from children with severe cerebral malaria pretreated with microvesicles containing anti-miR-155^34^. Others have reported elevated levels of miR-146a and miR-146b in exosomes secreted from LPS stimulated mouse macrophages^35^. Both miR-146a and miR-146b are upregulated in HUVECs upon exposure to IL1β along with increased protein expression of ICAM1, VCAM1 and CCL2^18^. The increase in both these miRNAs may be a negative feedback loop that, in fact, reduces inflammation in these cells by suppressing NF-κB and the MAPK pathways. Another study showed that LPS activates TLR4 that induces miR-146a through NF-κB. This up-regulated miR-146a targets tumor necrosis factor receptor-associated family 6 and interleukin-1 receptor-associated kinase 1 in the same TLR4/NF-κB pathway, keeping the innate immune response in check. This therefore implicates that prolonged exposure to circulating LPS and chronic viral infection elevating IFNα can dysregulate this mechanism leading to an inflammatory response^36^.

The brain function regulating miRNA-125a-5p can reduce cytokine induced monocyte migration in brain endothelium *in vitro* ^37^. miR-125a-5p improves the electrical resistance of the BBB by increasing the endothelial tight junction protein expression^23^. Studies have shown that the M2 macrophages known to be anti-inflammatory, highly express miR-125a-5p compared to M1 (proinflammatory) macrophages^38, 39^. miR-125a-5p overexpression suppressed LPS induced M1 phenotype expression, and promoted IL4 induced M2 expression in mice^38^. Another study showed that miR-125a-5p activates NF-κB in B-cell lymphoma cells by down-regulating tumor necrosis factor alpha-induced protein 3^16^. In addition, miR-125a-5p overexpression was reported to protect HBMECs from oxidized low density lipoprotein induced reactive oxygen species generation, nitric oxide production, senescence and apoptosis by targeting the Phosphoinositide 3-kinase/Protein kinase B/endothelial nitric-oxide synthase (PI3K/Akt/eNOS) and the epidermal growth factor receptor(EGFR)/ERK/p38 MAPK pathways^40^. Interestingly, miR-125a-5p overexpression was also shown to reduce leukocyte adhesion to HBMECs^40^. This correlates with our current observation that increased miR-125a-5p expression improves monocyte migration but not adherence to HBMECs. Up-regulation of miR-125a-5p in I/L stimulated monocytes and exosome treated HBMECs may be a defense response to protect the cells from the cytotoxic and inflammatory effect of I/L.

miR-222, is highly conserved in endothelial cells and is important for maintaining brain homeostasis^41^. In our current study, this miRNA was significantly down-regulated in I/L treated monocyte exosomes and in HBMECs. miR-222 protects the endothelium specifically by controlling its inflammatory activation and proliferation^15^. Decrease in the endothelial miR-221/222 cluster has been reported to contribute to various vascular disorders including coronary artery disease, heart failure, hypertension, obesity and atherosclerosis^14, 15^. In accordance with our findings, another report showed that HIV Tat treated HUVECs had significantly increased ICAM1 expression, which normalized with the overexpression of miR-222^19^. The ICAM1 increase and corresponding miR-222 decrease were NF-κB dependent^19^. Interestingly, a knock-down of miR-222 in HUVECs resulted in a decrease in miR-125^42, 43^. Given that miR-125a-5p is important for brain homeostasis by maintaining endothelial barrier function and immune cell balance, the associated decrease of both miR-125a-5p and miR-222 could possibly have worse physiological outcomes^33, 43^.

**Figure 7:**
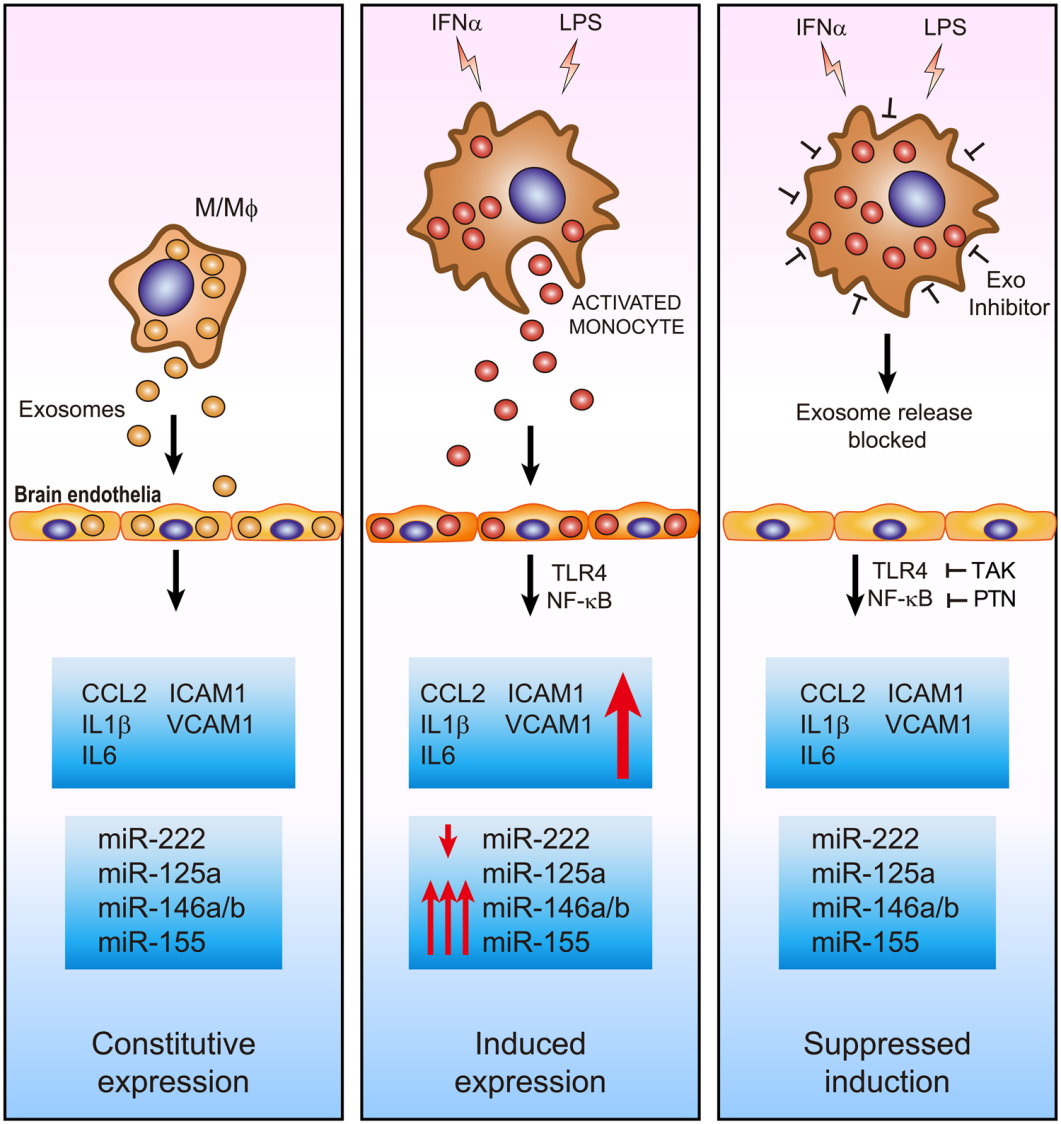
Schematic representing the key role of IFNα and LPS-activated monocyte-derived exosomes in brain endothelial stimulation. We propose that IFNα and LPS together cause significant changes in the monocyte exosome cargo, specifically miRNAs. These exosomes are taken up by the brain endothelial cells leading to damage in the form of abnormal upregulation of adhesion molecules, chemoattractants and pro-inflammatory cytokines. This mechanism is regulated by the TLR4/NF-κB pathway and involves dysregulated epigenetic operation by miRNAs known to be involved in these pathways. These miRNAs are constitutively expressed in endothelial cells (left panel). Stimulation of monocytes by IFNα and/or LPS changes the miRNA content within the exosomes and thereby the recipient endothelial cells (middle). Inhibiting the release of exosomes or TLR4 or NF-κB reverse this effect (right).

Cumulatively, based on these new findings and published reports, we conclude that the exosomes originating from monocytes influenced by exposure to LPS and IFNα carry dysregulated miRNA profiles (Fig. 7). These miRNAs are efficiently transferred to bystander brain endothelial cells via exosomes. These exosomes are crucial to monocyte chemotaxis since blocking exosome release mitigates the inflammatory responses. We propose that future efforts should be focused on combined gene therapies involving miRNA mimics for miR-222 and antagomirs for miR-155/miR-146 delivered through a cocktail of monocyte derived exosomes.

## Methods

### Monocyte isolation and activation

Leukocyte reduction chambers from normal human blood (n ≥ 3) were obtained from Blood Centers of the Pacific (San Francisco, CA, USA). The blood was diluted in PBS containing 2 mM EDTA within 2 h of collection and incubated with RosetteSep Monocyte Enrichment Cocktail (StemCell, Vancouver, BC, Canada). After 30 min, it was layered on Ficoll (GE Healthcare, Chicago, IL, USA) and centrifuged at 1200 g for 20 min. Monocytes settled at the PBS/Ficoll interface were collected and washed 2 times by centrifugation at 300 *g* and resuspended in PBS containing 2 mM EDTA. After a final spin at 300 *g*, the purified monocytes were resuspended in RPMI medium (Thermo Fisher Scientific Life Sciences, Waltham, MA, USA) containing 10% fetal bovine serum (FBS), 100 U/mL penicillin G and 100 μg/mL streptomycin. Cells (3 × 10^6^) were incubated in ultra-low attachment 6-well plates with rotation and stimulated with IFNα (100 U/mL) (PBL Assay Science, Piscataway, NJ, USA) for 4 h. In parallel, cells were also stimulated with LPS (1 ng/mL) (Sigma Aldrich, St. Louis, MO, USA) for 3 h. For combined IFNα and LPS (I/L) treatment, LPS was added after 1 h to the IFNα containing wells and further incubated for 3 h. After 4 h of stimulation, the monocytes were centrifuged at 300 *g* and resuspended in RPMI containing 10% FBS, antibiotics and 500 pg/mL macrophage colony stimulating factor. The cells were cultured for 24 h at 37 °C, 5% CO_2_.

### Exosome extraction and quantification

Exosomes from the 24 h stimulated monocytes were extracted using ExoQuick-TC reagent (System Biosciences, Palo Alto, CA, USA). The exosomes were quantified on a Nanosight LM10 instrument (Malvern Instruments, Malvern, UK) as described in our previous report^2^. The isolated exosome suspension in sterile PBS was added to HBMECs (1 × 10^4^ exosomes per 1 × 10^5^ cells) and cells incubated for 48 h before western blot analysis.

### Monocyte and endothelial cell coculture and migration study

Human brain microvascular endothelial cells (HBMECs, Cell Systems, Kirkland, WA, USA) were cultured in complete endothelial cell medium (Cell Systems) and used for experiments up to passage 9. Cells grown to 80% confluency in 24-well plates were used for the coculture assay. On the day of experiment, the medium was changed to complete recombinant serum free medium with 10% exosome free FBS (Cell Systems). Nonstimulated (NS) or activated monocytes were stained with calcein AM (1.5 μM) (Thermo Fisher Scientific) for 30 min at 37 °C, washed with PBS and re-suspended in RPMI containing 10% exosome-free FBS. They were seeded at a concentration of 4 × 10^5^ cells per FluoroBlok^TM^ insert of 8 μm pore size (Corning, Corning, Coring, NY, USA). The inserts were placed on top of the confluent HBMECs and exosome inhibitor GW4869 (10 μM)^44^ (Sigma-Aldrich) was immediately added in each insert per stimulation group (NS, IFNα, LPS, I/L). The monocyte migration toward HBMECs was quantified by measuring the increase in fluorescence at 485 nm excitation and 538 nm emission rate in the bottom chamber after 3 h of incubation at 37 °C.

### Exosome uptake by endothelial cells

The calcein AM stained monocytes present in the inserts described above, released calcein stained exosomes that were absorbed by the HBMECs cocultured in the bottom chamber. HBMECs grown on coverslips were mounted with DAPI containing Prolong Gold Antifade medium (Thermo Fisher Scientific) and visualized using Nikon Eclipse fluorescence microscope under 200X magnification for presence or absence of exosomes.

### qPCR

After 3 h of coculturing the IFNα and/or LPS stimulated monocytes with HBMECs, the HBMECs were lysed in Qiazol (Qiagen, Hilden, Germany). Total RNA was isolated using miRNeasy Mini Kit per manufacturer’s instructions (Qiagen). cDNA was produced using SuperScript^®^ III First-Strand Synthesis System (Thermo Fisher Scientific) for generating template for TaqMan^®^ qPCR assays *CCL2*, *ICAM1*, *VCAM1*, *IL6*, *IL1B* and *GAPDH* (Thermo Fisher Scientific). cDNA template for detecting miRNAs was generated using the Taqman MicroRNA Reverse Transcription Kit (Thermo Fisher Scientific) followed by qPCR using the TaqMan MircroRNA Assays for miR-146a, miR-146b, miR-155, miR-222 and miR-125a-5p with TaqMan Fast Advanced Master Mix for fluorescence detection. The ViiA 7 instrument (Thermo Fisher Scientific) was used for qPCR and the cycles were set up using comparative ΔΔC_t_ TaqMan Fast mode in the ViiA 7 RUO software Version 1.2.

### ELISA

Exosomes derived from monocytes activated with either IFNα, LPS or both were added to HBMECs cultured in exosome free FBS containing complete endothelial cell medium. After 48 h, cell supernatants were centrifuged at 3000 *g* and used for the detection of CCL2, IL6 and IL1β proteins by ELISA (R&D Systems, Minneapolis, MN, USA) using manufacturer’s instructions. The protein concentration was measured on a SpectraMax M5 Plate Reader (Molecular Devices, Sunnyvale CA, USA) using Softmax Pro5 software (Molecular Devices).

### Western Blot

HBMECs grown to 50% confluency were exposed to activated monocyte exosomes from 4 different blood donors in exosome free FBS containing complete endothelial cell medium for 48 h as described above followed by cell lysis using Mem-PER™ Eukaryotic Membrane Protein Extraction Reagent (Thermo Fisher Scientific). The extracted protein was measured using BCA reagent (Thermo Fisher Scientific) and separated at a concentration of 20 μg/20 μl on 12% Tris-glycine gel (Thermo Fisher Scientific). The gel was transferred to PVDF membrane (EMD Millipore, Billerica, MA, USA) and blocked with Odyssey Blocking Buffer (Li-Cor, Lincoln, NE, USA). The membranes were probed with ICAM1 and β-actin (Thermo Fisher Scientific) antibodies overnight at 4 °C, washed with PBS containing 0.1% Tween-20 followed by probing with IRDye 680 RD conjugated goat anti-rabbit and IRDye 800CW conjugated goat anti-mouse secondary antibodies (Li-Cor). The blots were visualized on Odyssey CLx Imager using the Image Studio software (version 4, Li-Cor). The same blots were stripped and probed for antibody against VCAM1 (Abcam, Cambridge, UK). In another set of experiments, exosomes from IFNα, LPS or I/L treated monocytes from de-identified donors were added to HBMECs for 1.5 h or 3 h followed by western blot for either TLR4 and MyD88 (Novus Biologicals, Littleton, CO, USA) or phosphorylated (p) NF-κB/p65 and total NF-κB (Cell Signaling Technologies, Danvers, MA, USA) respectively. All protein expressions were quantified on ImageJ software^45^ and normalized to β-actin.

### miRNA array

Human Taqman Low Density Array cards A and B (Thermo Fisher Scientific) were used for miRNA analysis. Total RNA was extracted from the exosomes of IFNα and/or LPS-treated monocytes using miRNeasy kit (Qiagen). One half microgram of total RNA was reverse-transcribed using Megaplex Pools and Taqman microRNA Reverse Transcription kit (Thermo Fisher Scientific) as described by the vendor. cDNA was analyzed using miRNA array card on a Viia7 qPCR instrument (Thermo Fisher Scientific). Data were normalized using global means method^46^.

### NF-κB and TLR4 inhibition

HBMECs seeded into 24-well plates were incubated for 48 h after which the medium was replaced with exosome depleted medium as described above. The cells were then treated with NF-κB inhibitor, parthenolide (PTN)^47^ or TLR4 inhibitor, TAK-242^48^ (both from InvivoGen, San Diego, CA, USA) for 90 min before incubation with activated monocyte exosomes for 24 h followed by qPCR for adhesion molecules, inflammatory markers and miRNAs.

### Statistics

Paired Student’s *t* test was used for all group comparisons. Data were analyzed using the R statistical programming software^49^ version 3.3.2. *P* < 0.05 was considered statistically significant. Multiple comparisons were adjusted using Benjamini and Hochberg’s method^50^. In the scatterplot graphs, each dot represents a data point and each horizontal bar represents the mean of the treatment group. Shading in the scatterplots indicates the range of the data and not the confidence interval. For trend test, Page’s trend test^51^ was chosen for repeated measures data and the analysis was performed using R package “crank” version 1.1.

## Electronic supplementary material

Dataset 1
