# Supplementary material for: Immune activated monocyte exosomes alter microRNAs in brain endothelial cells and initiate an inflammatory response through the TLR4/MyD88 pathway
Source: Sci Rep. 2017 Aug 30;7:9954. doi: 10.1038/s41598-017-10449-0 (PMC5577170; doi:10.1038/s41598-017-10449-0)
Supplement: Supplementary file 1 — Dataset 1 [file 41598_2017_10449_MOESM1_ESM.doc]

**Supplementary file**

**Immune activated monocyte exosomes alter microRNAs in brain endothelial cells and initiate an inflammatory response through the TLR4/MyD88 pathway**

**Pranjali Dalvi^1^, Bing Sun^1^, Norina Tang^1^, Lynn Pulliam^1,2*^**

^1^Department of Laboratory Medicine, Veterans Administration Medical Center, San Francisco, CA; ^2^Departments of Laboratory Medicine and Medicine, University of California, San Francisco, CA.

^1^Correspondence should be addressed to L.P. (email: [Lynn.Pulliam@ucsf.edu](mailto:Lynn.Pulliam@ucsf.edu))

N.T. current address is Sciogen LLC, Fremont, CA


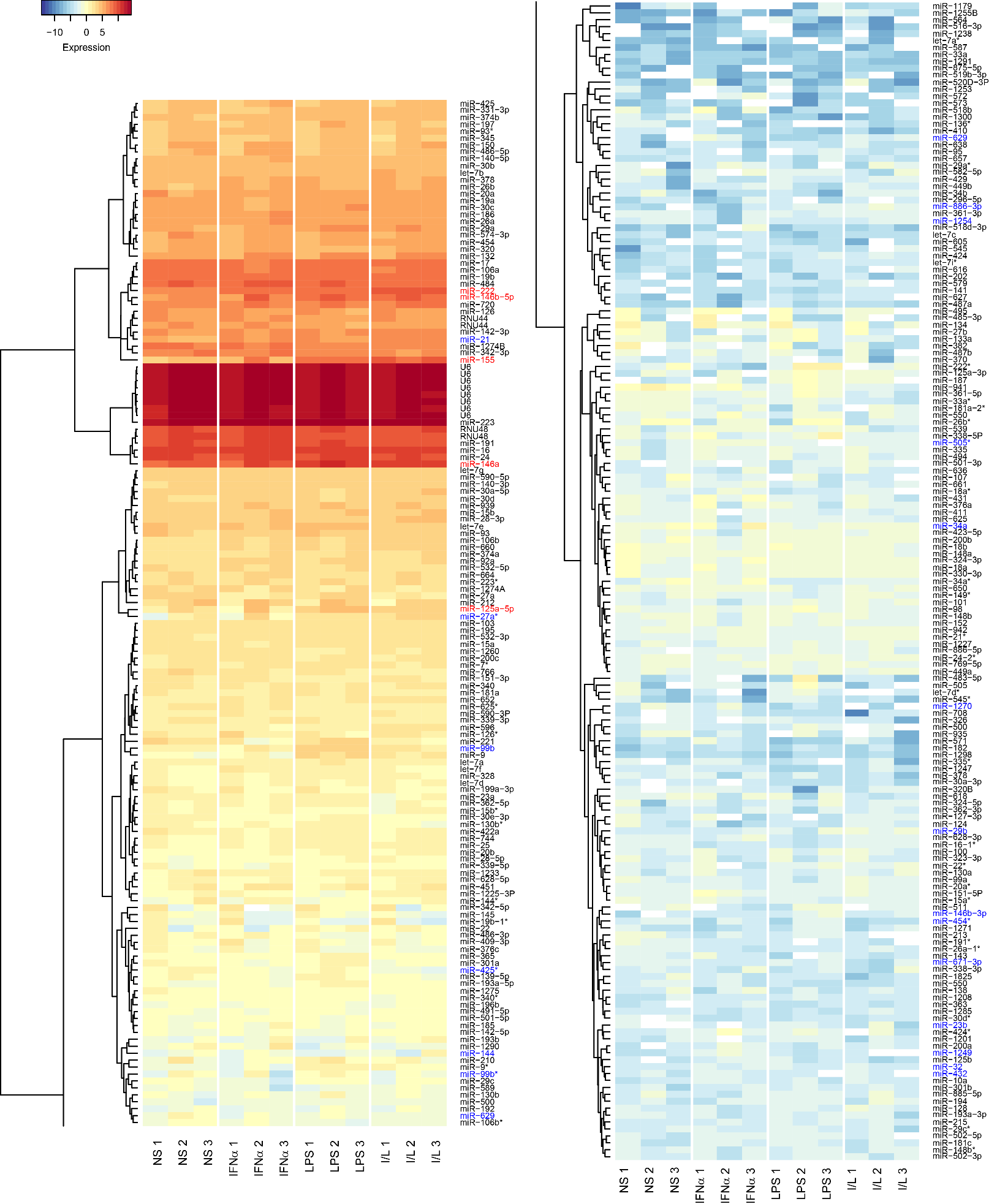


**Supplementary Figure S1. miRNAs are differentially regulated by IFNα and LPS stimulation of monocytes.** Heat map for miRNA arrays performed on normal human monocytes isolated from blood and stimulated with IFNα, LPS or both (I/L) (n=3). The significantly regulated miRs are presented in blue and red font. The miRs in red font are further analyzed in monocyte derived exosomes (Fig. 3B) and HBMECs (Fig. 4) due to their known involvement in inflammatory pathways.
